# Palliative care consultation in the last week of life and associated factors: a cross-sectional general population study

**DOI:** 10.1177/26323524241293818

**Published:** 2024-11-08

**Authors:** Susanna Böling, Hanna Gyllensten, My Engström, Emma Lundberg, Johan Berlin, Joakim Öhlén

**Affiliations:** Institute of Health and Care Sciences, Sahlgrenska Academy, University of Gothenburg, Arvid Wallgrens Backe, Box 457, Gothenburg 405 30, Sweden; Institute of Health and Care Sciences, Sahlgrenska Academy, University of Gothenburg, Gothenburg, Sweden; Institute of Health and Care Sciences, Sahlgrenska Academy, University of Gothenburg, Gothenburg, Sweden; Department of Surgery, Sahlgrenska University Hospital, Region Västra Götaland, Gothenburg, Sweden; Institute of Health and Care Sciences, Sahlgrenska Academy, University of Gothenburg, Gothenburg, Sweden; Department of Social and Behavioural Studies, University West, Trollhättan, Sweden; Institute of Health and Care Sciences, Sahlgrenska Academy, University of Gothenburg, Gothenburg, Sweden; Centre for Person-Centred Care, University of Gothenburg, Gothenburg, Sweden; Palliative Centre, Sahlgrenska University Hospital, Region Västra Götaland, Gothenburg, Sweden

**Keywords:** health services accessibility, palliative care, palliative medicine, palliative care team, patient care team, referral and consultation, terminal care

## Abstract

**Background:**

Knowledge of access to palliative care services, such as palliative care consultation teams, is crucial to identify areas of improvement for policy and practice. Research on general populations spanning all disease groups and multiple healthcare contexts is needed.

**Objective:**

The objective was to investigate the sociodemographic, disease- and care-related, and care structure-related factors associated with palliative care consultations for adult patients in the last week of life.

**Design:**

Cross-sectional, general population-level study based on linked Swedish national public authority registers and a national palliative care quality register.

**Methods:**

The study population included all adult patients deceased in Sweden between 2013 and 2019 and registered in the Swedish Register of Palliative Care, with an anticipated death, and not enrolled in specialised palliative care. Multivariable logistic regression analyses to investigate association with palliative care consultations.

**Results:**

In total, 8.2% of the 265,129 participants had received a palliative care consultation in the last week of life. The main multivariable analysis (Model 1) showed that those dying from neoplasms were more likely to receive a palliative care consultation (odds ratio (OR) 8.55, 95% CI 8.15–8.98) than those dying from circulatory diseases. Palliative care consultation was more likely with an increasing number of symptoms (OR 1.35, CI 1.32–1.37). Patients of old age and patients deceased in hospitals were less likely to receive a palliative care consultation. Moreover, factors such as educational attainment, healthcare region, living in a single-person household, and year of death were also associated with a palliative care consultation in the last week of life.

**Conclusion:**

Our findings show inequities in access to palliative care consultations in the last week of life. Considering changes to policy and clinical practice is motivated.

## Background

Inequities exist in regard to accessing palliative care^1,2^ despite it being recognised as a human right^3^ and an essential part of universal health coverage.^2,4^ This is a prevailing problem globally,^1,2^ regionally^5,6^ and locally.^7,8^ As a response, it is suggested that support, for example, through specialised palliative care consultations, is desirable in order to strengthen the integration of palliative care throughout all contexts and levels of care.^9^

Palliative care consultation services work in the intersection between specialised and non-specialised palliative care, trying to assist and strengthen palliative care for patients outside the specialised palliative care services.^10^ The services are often team-based and include a variety of competencies, such as medical, nursing, social and spiritual/religious.^11^ They assist in a range of problems and issues related to patient and family needs (e.g. symptom control) and coordination and improvement of care.^11,12^ Palliative care consultation services need not only be limited to the hospital context; rather, it is argued that there should be an opportunity for all healthcare services to consult specialised palliative care teams.^9^

Previous research indicates palliative care consultation services have a positive influence on the practice and perceptions of palliative care in acute care settings.^13^ Improvement has also been shown in patient symptoms, care satisfaction, advance care planning and reduction of needless interventions and costs.^11^ Furthermore, for nursing home residents, palliative care consultations seem to be associated with less acute care utilisation.^14,15^ From the evidence above, it seems palliative care consultations respond to the need for improved palliative care skills in non-specialised palliative care services, for example, hospitals.^16–18^ However, in nursing homes and primary care, a lack of consultation has been identified as a barrier towards palliative care provision.^19^

Factors associated with a palliative care consultation in specific contexts have been studied in previous research, for example, in various specialised medical settings^20–24^ and patient groups.^25^ Factors related to hospital characteristics^23–25^ and sociodemographic-^20,22,23,25^ and disease-^20,23–25^ attributes seem to be important for whether a palliative care consultation is offered or not. However, evidence is somewhat inconclusive, for example, regarding sex^22,24^ and ethnicity.^21,23–25^

Due to earlier research largely being limited to certain diagnosis groups or specific contexts,^22–24,26^ there is a need to examine wider populations spanning various healthcare settings and diseases. The objective of this study, therefore, was to investigate sociodemographic, disease- and care-related, and care structure-related factors associated with palliative care consultations for adult patients in the last week of life.

## Methods

### Study design

This study was retrospective cross-sectional, based on total population registry data for deceased persons in Sweden in the period 2013–2019 retrieved from national public authority registers and a national palliative care quality register in Sweden. The main outcome was palliative care consultations in the last week of life and their association with sociodemographic, disease- and care-related and care structure-related factors. The study was reported in accordance with the STROBE guideline^27^ and its extended version, RECORD.^28^

### Setting

Healthcare in Sweden encompasses all residents and is publicly financed, along with a minimal patient fee.^29^ Governance is shared by the state (overall policy), 21 regions (delivery and finance of healthcare services) and 290 municipalities (elderly care and care for the disabled, e.g. home care and nursing homes, for this study the latter including care for disabled).^30^ Each region and municipality has not only responsibility but also substantial freedom to organise its healthcare,^29^ which results in differences between regions.^30^ This also affects organisation of palliative care^30^ and access to specialised palliative care services,^18^ which are provided through specialised inpatient hospital units, hospices, specialised home care^30^ and consultation services. Palliative care consultation services are mainly based in specialised palliative care centres or units.^31^ Non-specialised palliative care is to be provided by all healthcare services as required.^32^ However, varied health system integration of palliative care has been reported.^6^

National clinical practice guidelines for palliative care^33^ were developed by healthcare professionals and published prior to the data, on which this study is based, was reported (the guidelines have been updated since^10,34^). Furthermore, a national guidance^31^ was published in 2013 by the National Board of Health and Welfare. These policy documents include a brief definition and recommendations for palliative care consultation teams and their practice.

### Study population

The study population was extracted from all registered deaths in Sweden from 2013 to 2019. It consisted of adults (⩾18 years) registered with the Swedish Register of Palliative Care^35^ who had an expected death or, where this was unsure, who were known to have received or not received a palliative care consultation in the last week of life, and who were not enrolled in a specialised palliative care service at the time of death (Supplemental File 1, eFigure 1). Data was retrieved from the following national registers: the Swedish National Cause of Death Register, the National Patient Register, the Total Population Register, the Education Register, the Historic Population Register, the Multi-generation Register and from the quality register; the Swedish Register of Palliative Care (Supplemental File 1, eTable 1). Register holder data linkage was based on personal identity number, replacing each number with a unique code before the data was made available to the researchers. The length of the study period was due to the national policy for palliative care being implemented from 2013 and that the COVID-19 pandemic was assumed to exert influence in other ways than in previous years.

**Table 1.** Descriptive summary of all variables, numbers (*n*) and proportion (%)^a^ of palliative care consultation for each subgroup on imputed data.

Variable	Total (*n* = 265,129)	Consultation: No (*n* = 243,317) (91,8%)	Consultation: Yes (*n* = 21,812) (8,2%)
Sex			
Male	115,771 (43.7%)	104,636 (43.0%)	11,135 (51.0%)
Female	149,358 (56.3%)	138,681 (57.0%)	10,677 (49.0%)
Age at death (continuous)			
	84.0 (10.2)	84.7 (9.8)	76.2 (11.8)
	86 (18; 113)	87 (18; 113)	77 (18; 106)
	*n* = 265,129	*n* = 243,317	*n* = 21,812
Age at death			
18–29	224 (0.1%)	175 (0.1%)	49 (0.2%)
30–39	411 (0.2%)	297 (0.1%)	114 (0.5%)
40–49	1397 (0.5%)	971 (0.4%)	426 (2.0%)
50–59	4755 (1.8%)	3451 (1.4%)	1304 (6.0%)
60–69	17,030 (6.4%)	13,404 (5.5%)	3626 (16.6%)
70–79	47,105 (17.8%)	40,289 (16.6%)	6816 (31.2%)
80–89	106,792 (40.3%)	99,644 (41.0%)	7148 (32.8%)
90+	87,415 (33.0%)	85,086 (35.0%)	2329 (10.7%)
Region of birth			
Sweden	238,934 (90.1%)	219,472 (90.2%)	19,462 (89.2%)
Nordic countries other than Sweden	13,512 (5.1%)	12,408 (5.1%)	1104 (5.1%)
EU28 other than Sweden and Nordic countries	6509 (2.5%)	5929 (2.4%)	580 (2.7%)
Outside Sweden, Nordic countries and EU28	6169 (2.3%)	5503 (2.3%)	666 (3.1%)
Educational attainment			
No formal or elementary education	124,297 (46.9%)	116,326 (47.8%)	7971 (36.5%)
Lower secondary education	19,669 (7.4%)	17,783 (7.3%)	1886 (8.6%)
Higher education	31,145 (11.7%)	28,041 (11.5%)	3104 (14.2%)
Higher secondary education	90,018 (34.0%)	81,167 (33.4%)	8851 (40.6%)
Marital status			
Married	78,984 (29.8%)	69,454 (28.5%)	9530 (43.7%)
Unmarried	26,494 (10.0%)	23,858 (9.8%)	2636 (12.1%)
Widow	121,884 (46.0%)	115,918 (47.6%)	5966 (27.4%)
Divorced	37,743 (14.2%)	34,067 (14.0%)	3676 (16.9%)
Living in a single-person household			
Multi-person household	110,900 (41.8%)	98,424 (40.5%)	12,476 (57.2%)
Single-person household	154,229 (58.2%)	144,893 (59.5%)	9336 (42.8%)
Have children under the age of 18			
No children under 18	259,732 (98.0%)	238,709 (98.1%)	21,023 (96.4%)
Have children under 18	5397 (2.0%)	4608 (1.9%)	789 (3.6%)
Living situation			
Owned residence	120,378 (45.4%)	106,602 (43.8%)	13,776 (63.2%)
Rented residence	59,839 (22.6%)	53,970 (22.2%)	5869 (26.9%)
Nursing home	79,034 (29.8%)	77,258 (31.8%)	1776 (8.1%)
Other	5878 (2.2%)	5487 (2.3%)	391 (1.8%)
Residing in an urban area			
Not residing in an urban area	28,283 (10.7%)	24,549 (10.1%)	3734 (17.1%)
Residing in an urban area	236,824 (89.3%)	218,750 (89.9%)	18,074 (82.9%)
Underlying cause of death			
Diseases of the circulatory system	89,303 (33.7%)	87,107 (35.8%)	2196 (10.1%)
Neoplasms	63,619 (24.0%)	47,028 (19.3%)	16,591 (76.1%)
Diseases of the digestive system	7481 (2.8%)	7122 (2.9%)	359 (1.6%)
Diseases of the nervous system	7289 (2.7%)	6866 (2.8%)	423 (1.9%)
Diseases of the respiratory system	19,497 (7.4%)	18,845 (7.7%)	652 (3.0%)
Endocrine, nutritional and metabolic diseases	7657 (2.9%)	7399 (3.0%)	258 (1.2%)
Infectious diseases	7281 (2.7%)	7065 (2.9%)	216 (1.0%)
Dementia^b^	45,505 (17.2%)	44,925 (18.5%)	580 (2.7%)
Other	17,497 (6.6%)	16,960 (7.0%)	537 (2.5%)
Place of death			
Hospital	95,893 (36.2%)	87,845 (36.1%)	8048 (36.9%)
Nursing home	149,044 (56.2%)	140,210 (57.6%)	8834 (40.5%)
Home	19,746 (7.4%)	14,908 (6.1%)	4838 (22.2%)
Other place or unknown	446 (0.2%)	354 (0.1%)	92 (0.4%)
Number of hospital transfers in last month of life (continuous)			
	0.748 (0.917)	0.729 (0.912)	0.953 (0.946)
	1 (0; 10)	0 (0; 10)	1 (0; 9)
	*n* = 265,129	*n* = 243,317	*n* = 21,812
Number of hospital transfers in the last month of life (categorical)			
None	130,924 (49.4%)	123,001 (50.6%)	7923 (36.3%)
One transfer	87,195 (32.9%)	78,404 (32.2%)	8791 (40.3%)
Two transfers	34,103 (12.9%)	30,359 (12.5%)	3744 (17.2%)
Three or more transfers	12,907 (4.9%)	11,553 (4.7%)	1354 (6.2%)
Number of days in the reporting care service (continuous)			
	540 (939)	572 (959)	182.4 (549.3)
	60 (0; 38,128)	80 (0; 38,128)	21 (0; 12,934)
	*n* = 264,968	*n* = 243,168	*n* = 21,800
Number of days in the reporting care service (categorical)			
0–2	27,616 (10.4%)	25,944 (10.7%)	1672 (7.7%)
3–7	35,956 (13.6%)	32,612 (13.4%)	3344 (15.3%)
8–30	54,004 (20.4%)	46,158 (19.0%)	7846 (36.0%)
31–182	35,535 (13.4%)	30,187 (12.4%)	5348 (24.5%)
183–365	16,615 (6.3%)	15,484 (6.4%)	1131 (5.2%)
366–	95,242 (35.9%)	92,783 (38.2%)	2459 (11.3%)
Symptom presence in the last week of life			
No reported symptoms	27,933 (10.5%)	27,081 (11.1%)	852 (3.9%)
One symptom	52,595 (19.8%)	49,899 (20.5%)	2696 (12.4%)
Two symptoms	76,731 (28.9%)	70,997 (29.2%)	5734 (26.3%)
Three or more symptoms	107,870 (40.7%)	95,340 (39.2%)	12,530 (57.4%)
Healthcare region			
South region	47,533 (17.9%)	43,591 (17.9%)	3942 (18.1%)
Southeast region	36,535 (13.8%)	32,635 (13.4%)	3900 (17.9%)
West region	52,921 (20.0%)	47,278 (19.4%)	5643 (25.9%)
Stockholm region	33,324 (12.6%)	32,394 (13.3%)	930 (4.3%)
Uppsala-Örebro region	65,650 (24.8%)	60,626 (24.9%)	5024 (23.0%)
North region	29,144 (11.0%)	26,775 (11.0%)	2369 (10.9%)
Year of death			
2013	38,251 (14.4%)	35,294 (14.5%)	2957 (13.6%)
2014	39,128 (14.8%)	35,976 (14.8%)	3152 (14.5%)
2015	39,051 (14.7%)	35,905 (14.8%)	3146 (14.4%)
2016	38,632 (14.6%)	35,457 (14.6%)	3175 (14.6%)
2017	38,380 (14.5%)	35,123 (14.4%)	3257 (14.9%)
2018	36,462 (13.8%)	33,379 (13.7%)	3083 (14.1%)
2019	35,225 (13.3%)	32,183 (13.2%)	3042 (13.9%)

For categorical variables, numbers (*n*) and proportion (%) are presented. For continuous variables, mean (SD)/median (min; max)/*n*= are presented.

a Proportion (%) represents column percentage.

b Including senility.

### Variables and data sources

The main outcome, ‘consultation with a palliative care consultation service’ (yes/no), and variables defining the population (Supplemental File 2) were retrieved from either the Swedish Register of Palliative Care or the Cause of Death register. Enrolled in a specialised palliative care service at the time of death (yes/no) was based on the reported place of death in the Swedish Register of Palliative Care. A free-text option, ‘other’ (place), was categorised manually by the first author. For expected death (yes/no/don’t know), ‘don’t know’ was included in the ‘yes’ category, as this was considered a situation where the uncertainty surrounding the death may require a palliative care consultation. The outcome variable, ‘consultation with a palliative care consultation service’, was based on a question in the Swedish Register of Palliative Care: ‘*Were specialists outside the team/ward consulted concerning the person’s symptom relief during the last week of life?*’^36^ with multiple-choice options for answers, for example, ‘*yes, palliative team*’ or ‘*don’t know*’. We excluded patients whom the care provider answered ‘*don’t know*’. The outcome variable reflects the patient’s last week of life, and palliative care consultations that may have occurred prior to this period were therefore neither included in this study nor reported in the register.

Variables examined for association with the dependent variable were divided into sociodemographic characteristics, disease- and care-related and care structure-related factors. Variables, coding and source registers are presented in Supplemental File 2.

### Statistical analysis

**Figure 1.**
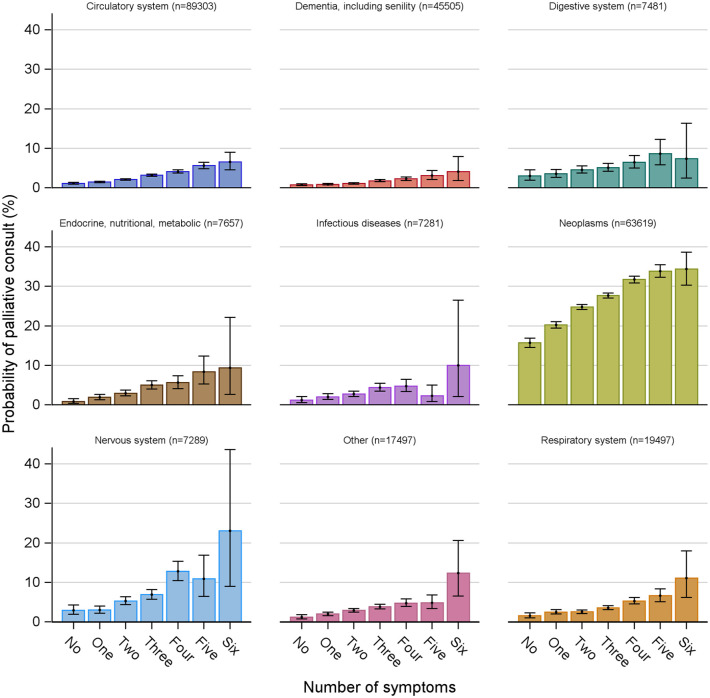
Probability of palliative care consultation for each diagnosis group related to the reported number of symptoms in the last week of life.

Descriptive statistics including numbers and proportion of palliative care consultation for each variable were calculated (Table 1). Association with the dependent variable ‘consultation with a palliative care consultation service’ (yes/no) was tested in univariable analyses for each explanatory variable (Table 2). Multivariable logistic regression was used to test the association between the dependent variable and a theory-driven core set of explanatory variables which formed the main analysis (Multivariable Model 1, Table 3). Each group of explanatory variables (sociodemographic characteristics, disease- and care-related factors and care structure-related factors) was tested separately and all together with multivariable logistic regression (Supplemental File 1, eTables 2–5). Results were presented in odds ratio (OR) with a 95% confidence interval (CI). Area under the ROC curve (AUC) was calculated for model goodness of fit. Additionally, a selection of best predictor variables was calculated based on the Akaike Information Criterion (best seven variables model, Multivariable Model 2, Table 4). Missing data in four explanatory variables (Supplemental File 2) with the greatest number of missing data were imputed with stochastic imputation, using Fully Conditional Specification with prespecified seed 4889. All statistical analyses were performed on imputed data, and sensitivity analyses were made on all available data (Supplemental File 1, eTables 6 and 7). A *p*-value of <0.01 was considered significant. Based on the preliminary results, an analysis was conducted of palliative care consultation probability for each diagnosis group related to the reported number of symptoms in the last week of life (Figure 1). IBM SPSS Statistics 28.0.1.0, IBM Corporation, was used for preparation of variables. SAS 9.4, SAS Institute Inc., was also used for the preparation of variables as well as statistical analyses.

**Table 2.** Univariable logistic regression model with all variables – associated factors with the consultation of a palliative care consultation service in the last week of life on imputed data.

Variable	*n* missing	*n* (%) of event	OR (95% CI)	*p*-Value	Area under ROC curve (95% CI)
			Consultation with a palliative care consultation service		
Sex	0				
Male		11,135 (9.6%)	1.00		
Female		10,677 (7.1%)	0.72 (0.70–0.74)	<0.0001	0.54 (0.54–0.54)
Age at death continuous (OR per 10 units)	0				
18–113		21,812 (8.2%)	0.53 (0.52–0.53)	<0.0001	0.73 (0.72–0.73)
Age at death	0				
80–89		7148 (6.7%)	1.00	<0.0001***	
18–29		49 (21.9%)	3.90 (2.84–5.36)	<0.0001	
30–39		114 (27.7%)	5.35 (4.31–6.65)	<0.0001	
40–49		426 (30.5%)	6.12 (5.44–6.87)	<0.0001	
50–59		1304 (27.4%)	5.27 (4.92–5.64)	<0.0001	
60–69		3626 (21.3%)	3.77 (3.61–3.94)	<0.0001	
70–79		6816 (14.5%)	2.36 (2.28–2.44)	<0.0001	
90+		2329 (2.7%)	0.38 (0.36–0.40)	<0.0001	0.71 (0.70–0.71)
Region of birth	5				
Sweden		19,462 (8.1%)	1.00	<0.0001***	
Nordic countries outside Sweden		1104 (8.2%)	1.00 (0.94–1.07)	0.92	
EU28		580 (8.9%)	1.10 (1.01–1.20)	0.026	
Outside EU28 and Nordic countries		666 (10.8%)	1.37 (1.26–1.48)	<0.0001	0.51 (0.50–0.51)
Educational attainment	0				
Higher secondary education		8851 (9.8%)	1.00	<0.0001***	
No formal or elementary education		7971 (6.4%)	0.63 (0.61–0.65)	<0.0001	
Lower secondary education		1886 (9.6%)	0.97 (0.92–1.02)	0.30	
Higher education		3104 (10.0%)	1.02 (0.97–1.06)	0.49	0.56 (0.55–0.56)
Marital status	24				
Married		9530 (12.1%)	1.00	<0.0001***	
Unmarried		2636 (9.9%)	0.81 (0.77–0.84)	<0.0001	
Widow		5966 (4.9%)	0.38 (0.36–0.39)	<0.0001	
Divorced		3676 (9.7%)	0.79 (0.76–0.82)	<0.0001	0.61 (0.61–0.62)
Living in a single-person household	0				
Multi-person household		12,476 (11.2%)	1.00		
Single-person household		9336 (6.1%)	0.51 (0.49–0.52)	<0.0001	0.58 (0.58–0.59)
Have children under the age of 18	0				
No children under 18		21,023 (8.1%)	1.00		
Children under 18		789 (14.6%)	1.94 (1.80–2.10)	<0.0001	0.51 (0.51–0.51)
Living situation	0				
Owned residence		13,776 (11.4%)	1.00	<0.0001***	
Rented residence		5869 (9.8%)	0.84 (0.81–0.87)	<0.0001	
Nursing home		1776 (2.2%)	0.18 (0.17–0.19)	<0.0001	
Other		391 (6.7%)	0.55 (0.50–0.61)	<0.0001	0.63 (0.63–0.64)
Residing in an urban area	22				
Not residing in an urban area		3734 (13.2%)	1.00		
Residing in an urban area		18,074 (7.6%)	0.54 (0.52–0.56)	<0.0001	0.54 (0.53–0.54)
Underlying cause of death	0				
Diseases of the circulatory system		2196 (2.5%)	1.00	<0.0001***	
Neoplasms		16,591 (26.1%)	13.99 (13.37–14.65)	<0.0001	
Diseases of the digestive system		359 (4.8%)	2.00 (1.78–2.24)	<0.0001	
Diseases of the nervous system		423 (5.8%)	2.44 (2.20–2.72)	<0.0001	
Diseases of the respiratory system		652 (3.3%)	1.37 (1.26–1.50)	<0.0001	
Endocrine, nutritional and metabolic diseases		258 (3.4%)	1.38 (1.21–1.58)	<0.0001	
Infectious diseases		216 (3.0%)	1.21 (1.05–1.40)	0.0077	
Dementia^a^		580 (1.3%)	0.51 (0.47–0.56)	<0.0001	
Other		537 (3.1%)	1.26 (1.14–1.38)	<0.0001	0.80 (0.80–0.81)
Place of death	0				
Hospital		8048 (8.4%)	1.00	<0.0001***	
Nursing home		8834 (5.9%)	0.69 (0.67–0.71)	<0.0001	
Home		4838 (24.5%)	3.54 (3.40–3.69)	<0.0001	
Other place or unknown		92 (20.6%)	2.84 (2.25–3.57)	<0.0001	0.61 (0.61–0.62)
Number of hospital transfers last month of life (continuous)	0				
0 to <1		7923 (6.1%)			
1–10		13,889 (10.3%)	1.27 (1.25–1.28)	<0.0001	0.57 (0.57–0.58)
Number of hospital transfers last month of life (categorical)	0				
None		7923 (6.1%)	1.00	<0.0001***	
One transfer		8791 (10.1%)	1.74 (1.69–1.80)	<0.0001	
Two transfers		3744 (11.0%)	1.91 (1.84–1.99)	<0.0001	
Three or more transfers		1354 (10.5%)	1.82 (1.71–1.93)	<0.0001	0.57 (0.57–0.58)
Number of days in the reporting care service (OR per 30 units)	161				
0 to <14		8282 (9.3%)			
14 to <454		11,408 (13.0%)			
454 to 38,128		2110 (2.4%)	0.97 (0.97–0.97)	<0.0001	0.61 (0.60–0.61)
Number of days in the reporting care service	161				
0–2		1672 (6.1%)			
3–7		3344 (9.3%)			
8–30		7846 (14.5%)			
31–182		5348 (15.0%)			
183–365		1131 (6.8%)			
366–		2459 (2.6%)	0.81 (0.80–0.82)	<0.0001	0.61 (0.60–0.61)
Symptom presence in the last week of life	0				
No reported symptoms		852 (3.1%)			
One symptom		2696 (5.1%)			
Two symptoms		5734 (7.5%)			
Three or more symptoms		12,530 (11.6%)	1.59 (1.56–1.61)	<0.0001	0.61 (0.61–0.62)
Healthcare region	22				
South region		3942 (8.3%)	1.00	<0.0001***	
Southeast region		3900 (10.7%)	1.32 (1.26–1.38)	<0.0001	
West region		5643 (10.7%)	1.32 (1.26–1.38)	<0.0001	
Stockholm region		930 (2.8%)	0.32 (0.30–0.34)	<0.0001	
Uppsala-Örebro region		5024 (7.7%)	0.92 (0.88–0.96)	<0.0001	
North region		2369 (8.1%)	0.98 (0.93–1.03)	0.42	0.58 (0.58–0.58)
Year of death (continuous)	0				
2013		2957 (7.7%)			
2014		3152 (8.1%)			
2015		3146 (8.1%)			
2016		3175 (8.2%)			
2017		3257 (8.5%)			
2018		3083 (8.5%)			
2019		3042 (8.6%)	1.02 (1.01–1.03)	<0.0001	0.51 (0.51–0.51)

All tests are performed with univariable logistic regression. *p*-Values, OR and area under ROC curve are based on original values and not on stratified groups. OR is the ratio for the odds for an increase of the predictor of 1 unit.

a Including senility.

*** *p*-Value for the entire effect/factor/variable.

CI, confidence interval; OR, odds ratio.

**Table 3.** Multivariable Model 1. Multivariable logistic regression model – main analysis with a core set of explanatory variables – factors associated with consultation of a palliative care consultation service in the last week of life on imputed data.

Variable	OR (95% CI)	Pr > chi-square	Variable *p*-value
Age at death			<0.0001
80–89	1		
18–29	2.18 (1.52–3.12)	<0.0001	
30–39	2.54 (1.99–3.23)	<0.0001	
40–49	2.68 (2.35–3.06)	<0.0001	
50–59	2.37 (2.19–2.56)	<0.0001	
60–69	1.90 (1.80–2.00)	<0.0001	
70–79	1.52 (1.46–1.58)	<0.0001	
90+	0.56 (0.53–0.59)	<0.0001	
Year of death	1.03 (1.02–1.04)	<0.0001	<0.0001
Place of death			<0.0001
Hospital	1		
Home	2.85 (2.73–2.99)	<0.0001	
Nursing home	1.39 (1.34–1.44)	<0.0001	
Other place or unknown	2.39 (1.85–3.10)	<0.0001	
Sex			0.12
Male	1		
Female	1.03 (0.99–1.06)	0.12	
Region of birth			0.31
Sweden	1		
Nordic countries outside Sweden	1.01 (0.94–1.08)	0.82	
EU28	1.08 (0.98–1.19)	0.12	
Outside EU28 and Nordic countries	0.96 (0.87–1.05)	0.34	
Underlying cause of death			<0.0001
Diseases of the circulatory system	1		
Neoplasms	8.55 (8.15–8.98)	<0.0001	
Infectious diseases	1.22 (1.06–1.41)	0.0067	
Endocrine, nutritional and metabolic diseases	1.14 (1.00–1.30)	0.049	
Diseases of the respiratory system	1.24 (1.13–1.35)	<0.0001	
Diseases of the nervous system	1.63 (1.46–1.82)	<0.0001	
Diseases of the digestive system	1.63 (1.46–1.84)	<0.0001	
Dementia^a^	0.52 (0.48–0.58)	<0.0001	
Other	1.22 (1.10–1.34)	<0.0001	
Educational attainment			0.0010
Higher secondary education	1		
No formal or elementary education	0.94 (0.91–0.98)	0.0015	
Lower secondary education	0.92 (0.87–0.97)	0.0045	
Higher education	1.00 (0.95–1.05)	0.95	
Healthcare region			<0.0001
South region	1		
Southeast region	1.28 (1.22–1.35)	<0.0001	
West region	1.25 (1.20–1.31)	<0.0001	
Stockholm region	0.46 (0.43–0.50)	<0.0001	
Uppsala-Örebro region	0.88 (0.84–0.93)	<0.0001	
North region	1.12 (1.06–1.19)	0.0001	
Living in single-person household			<0.0001
Multi-person household	1		
Single-person household	0.86 (0.83–0.89)	<0.0001	
Symptom presence in the last week of life			<0.0001
No reported symptoms			
One symptom			
Two symptoms			
Three or more symptoms	1.35 (1.32–1.37)	<0.0001	

Area under ROC curve with 95% CI for multivariable model = 0.85 (0.84–0.85).

a Including senility.

CI, confidence interval; OR, odds ratio.

**Table 4.** Multivariable Model 2. Best model with 7 variables based on AIC – factors associated with consultation of a palliative care consultation service in the last week of life.

Variable	OR (95% CI)	Pr > chi-square	Variable *p*-value
Age at death			<0.0001
80–89	1		
18–29	2.30 (1.62–3.29)	<0.0001	
30–39	2.57 (2.02–3.28)	<0.0001	
40–49	2.72 (2.39–3.10)	<0.0001	
50–59	2.37 (2.20–2.56)	<0.0001	
60–69	1.87 (1.78–1.96)	<0.0001	
70–79	1.48 (1.43–1.54)	<0.0001	
90+	0.60 (0.57–0.63)	<0.0001	
Living in a single-person household			0.0084
Multi-person household	1		
Single-person household	0.96 (0.92–0.99)	0.0084	
Symptom presence in the last week of life			<0.0001
No reported symptoms			
One symptom			
Two symptoms			
Three or more symptoms	1.36 (1.34–1.39)	<0.0001	
Number of days in the reporting care service (categorical)			<0.0001
0–2			
3–7			
8–30			
31–182			
183–365			
366–	0.89 (0.88–0.90)	<0.0001	
Living situation			<0.0001
Owned residence	1		
Rented residence	0.94 (0.91–0.98)	0.0018	
Nursing home	0.57 (0.54–0.60)	<0.0001	
Other	0.82 (0.73–0.92)	0.0006	
Underlying cause of death			<0.0001
Diseases of the circulatory system	1		
Neoplasms	7.67 (7.31–8.06)	<0.0001	
Diseases of the digestive system	1.52 (1.35–1.71)	<0.0001	
Diseases of the nervous system	1.72 (1.54–1.92)	<0.0001	
Diseases of the respiratory system	1.21 (1.11–1.32)	<0.0001	
Endocrine, nutritional and metabolic diseases	1.19 (1.04–1.36)	0.0113	
Infectious diseases	1.20 (1.04–1.39)	0.0114	
Dementia^a^	0.60 (0.54–0.66)	<0.0001	
Other	1.21 (1.10–1.33)	0.0001	
Place of death			<0.0001
Hospital	1		
Nursing home	1.87 (1.79–1.95)	<0.0001	
Home	3.88 (3.69–4.09)	<0.0001	
Other place or unknown	2.58 (2.00–3.33)	<0.0001	

161 observations were deleted due to missing values for the response or explanatory variables. Area under ROC curve for multivariable model = 0.85

a Including senility.

AIC, Akaike Information Criterion; CI, confidence interval; OR, odds ratio.

## Results

A total of 265,129 patients were included in the study (Table 1 and Supplemental File 1, eFigure 1). Of this total, 8.2% (21,812) had a consultation with a palliative care consultation service during their last week of life. Although the average age at death was 84.0 years for the full sample, it was lower among those who had received a palliative care consultation (76.2 years; Table 1). In the full sample, diseases of the circulatory system were the most common underlying cause of death (33.7%) followed by neoplasms (24.0%) and dementia (17.2%). Among patients who received a palliative care consultation, neoplasm was the leading diagnosis (76.1%), diseases of the circulatory system constituted a tenth (10.1%) and dementia even less (2.7%). Nursing home was the leading place of death in the full sample (56.2%; Table 1) and had the lowest proportion of palliative care consultations (5.9%; Table 2). Of the study population, 40.7% were reported to have three or more symptoms in the last week of life (Table 1). This was also true for 57.4% of those who had a palliative care consultation. In comparison with the source population, the study population was older and had a higher percentage deceased in nursing homes and dying from dementia (numbers not reported).

### Associated factors with a palliative care consultation in the last week of life

Patients who died from neoplasms (OR 8.55, 95% CI 8.15–8.98) were most likely to have received a palliative care consultation in their last week of life compared to patients who died from diseases of the circulatory system (Table 3, Multivariable Model 1, AUC 0.85, 95% CI 0.84–0.85). For all other causes of death, the likelihood of receiving a palliative care consultation was higher than for diseases of the circulatory system, except for dementia (OR 0.52, 95% CI 0.48–0.58). Endocrine, nutritional and metabolic diseases were not significant in the theory-driven model (Table 3, Multivariable Model 1). Compared to people aged 80–89, the likelihood of receiving a palliative care consultation increased through the age group 40–49 and then decreased for each increment in the age group. A higher number of symptoms in the last week of life (OR 1.35, 95% CI 1.32–1.37) was associated with an increased chance of receiving a palliative care consultation. Association between a palliative care consultation and place of death was also seen, with the home being the most likely place to have received a palliative care consultation (OR 2.85, 95% CI 2.73–2.99) compared to death in hospital, followed by other place or unknown (2.39, 95% CI 1.85–3.10) and nursing home (OR 1.39, 95% CI 1.34–1.44). More recent years of death slightly increased the likelihood of a palliative care consultation (OR 1.03, 95% CI 1.02–1.04). Among healthcare regions, the capital region (Stockholm region) stood out as having the least likelihood for a patient to receive a palliative care consultation (OR 0.46, 95% CI 0.43–0.50) compared to the South region. People living in a single-person household were less likely to receive a palliative care consultation (OR 0.86, 95% CI 0.83–0.89), although this relationship was inconclusive between models. People with no formal or elementary education (OR 0.94, 95% CI 0.91–0.98) and lower secondary education (OR 0.92, 95% CI 0.87–0.97) were less likely to receive a palliative care consultation in the last week of life compared to people with higher secondary education (Table 3). However, these relationships were inconclusive between models.

Sex was not significantly associated with a palliative care consultation in the theory-driven model (Table 3), and neither was the region of birth. No major differences were seen between the theory-driven model on imputed and all available data.

The best predictive model included the variables age at death, place of death, underlying cause of death, symptom presence in the last week of life, living in a single-person household, living situation and number of days in the reporting care service (Table 4, Multivariable Model 2, AUC 0.85). In addition to the variables associated with palliative care consultation present in the theory-driven model, the variable number of days in the reporting care service was included, whereby more days decreased the likelihood of a palliative care consultation (OR 0.89, 95% CI 0.88–0.90). Living situation was also included, whereby all categories compared to owned residence decreased the likelihood. However, only nursing home was significant in the model with all variables (Supplemental File 1, eTable 5). There were no major differences between the variables overlapping between the theory-driven model and the best predictive model.

Marital status, having children under the age of 18, residing in an urban area and the number of hospital transfers were also tested (Supplemental File 1, eTables 2, 3 and 5). It is worth mentioning that residing in an urban area decreased the likelihood of a palliative care consultation, while one or more hospital transfers increased the likelihood. Moreover, being unmarried presented a lower likelihood of receiving a palliative care consultation than being married.

The analysis of probability for a palliative care consultation related to the diagnosis group and reported number of symptoms (Figure 1) shows that, overall, the probability of a palliative care consultation increases for all causes of death, as the number of symptoms increases in the last week of life.

## Discussion

### Main findings

Our findings made it evident that long-standing clinical structures and perceptions still exist that favour patients with neoplasms for access to palliative care consultations. In contrast, dying in a hospital, living in the capital region, as well as factors related to older people reduced the likelihood of palliative care consultations in the last week of life. These findings imply inequities in access. However, a larger number of symptoms increased the likelihood of consultations, which indicates that patients with the greatest needs receive consultations. Hence, our findings show that sociodemographic factors, as well as disease- and care-related, and care structure-related factors, are associated with patients’ receiving a palliative care consultation in their last week of life.

### What this study adds and implications for practice

Most studies of access to palliative care consultation services are concentrated on specific disease groups^25^ or settings.^20–24^ Our study adds a wider perspective, spanning all disease groups and multiple care contexts, and leaves us with the opportunity to compare these different features.

In line with the prevailing evidence regarding access to specialised palliative care,^37,38^ patients dying from neoplasms stood out as most likely to have had a palliative care consultation performed in the last week of life compared to other diseases. Since palliative care consultation services operate in the intersection between specialised and non-specialised palliative care, this should impact non-specialised palliative care provision for other disease groups. This oblique allocation of palliative competence calls for structural reforms within healthcare, with further collaboration between specialised palliative care and specialities other than cancer care to improve palliative care integration and access.

The only group with lower odds of receiving palliative care consultations than circulatory diseases was dementia, a disease group often associated with old age and care in nursing homes. Moreover, our findings show that from the ages of 50–59, the likelihood of receiving a consultation decreased. This evidence suggests that older people have less access to palliative care consultations than younger ones, despite evidence from UK hospital settings suggesting that older people (without cancer) are the largest patient group with palliative care needs.^16^ Our finding largely resonates with the overall European and Swedish context where integration of palliative care in long-term care facilities seems limited.^6^ Nevertheless, although only 5.9% of those deceased in nursing homes received a palliative care consultation, this group was more likely to receive a palliative care consultation than people who died in hospitals. There is a possibility that some of the older people included in the study were under the care of a geriatrician or another hospital specialist. However, those residing in nursing homes typically had a general practitioner as their responsible physician. In either case, there may be situations where specialised palliative care knowledge is beneficial for these patients, for example in managing severe symptom distress. This potential need is highlighted in previous research, which shows an association between older age and a poorer quality of end-of-life care (including fewer palliative care consultations) for patients dying from neoplasm.^39^ Further research on how clinicians and society as a whole view end-of-life among older people is warranted to disentangle reasons for possible inequalities in access to palliative care.

Our study emphasises the association between healthcare region and access to palliative care consultations, with the capital region appearing to be the healthcare region with the lowest odds of patients receiving a palliative care consultation. The regional responsibility of specialised palliative care provision and organisation^30^ seems to influence access to palliative care consultations and puts into question whether Sweden has succeeded with its objective of healthcare on ‘equal terms’ for all, which is established by law.^40^ Nevertheless, varying demographic and geographical prerequisites may require adapted care structures. The capital region, for example, has a relatively well-developed specialised palliative home care capacity in comparison with other regions who to a greater extent organise their palliative home care through non-specialised home care, supported by palliative care consultation services. The latter organisation of palliative care could be a contributing factor to our finding that palliative care consultations are more likely for patients that die in their homes. Moreover, our study indicates there was limited access to palliative care consultations in hospitals compared to other healthcare services. Efforts to ensure the availability of and access to palliative care consultation services across all healthcare services are needed.

Greater symptom burden was associated with a higher likelihood of receiving palliative care consultations, which was somewhat expected, since symptom management is a core competence of palliative care consultation services. Nevertheless, some caution should be taken when interpreting these findings due to how the question regarding consultation is phrased in the register (see ‘Variables and data sources’), which could bias the importance of symptom presence. Furthermore, neither symptom severity nor problems like, for example, existential distress and complicated family situations, are included in our study. Factors that possibly relate to the likelihood of a consultation.

In the context of residential care homes, Andersson et al.^41^ found that consultations were conversely associated with relieved pain. However, if palliative care consultation services are provided to patients with complex symptoms during their last week of life, an unfortunate consequence could be that the consultation does not achieve its aim for all symptoms. Although there in some cases may have been unexpected fatal events leading to consultation in the last week of life, this suggests that referral to palliative care consultation services would have been desirable much earlier in the disease trajectory.

### Strengths and weaknesses

A strength of this study is that the study group represents the general population of a country, regardless of sociodemographic, economic or disease background. Sweden has a long-standing tradition^42^ of collecting reliable population statistics^43–45^ which strengthens the validity of the study. Nevertheless, the data was not gathered specifically for this study, and errors may have occurred during the collection process. Moreover, a cross-sectional design implies difficulties in determining the directionality of the associations, and other potentially important influencing factors may have been omitted. Although caution should be taken when transferring these findings to other contexts, similarities may be found in countries with comparable societal, healthcare and palliative care features.

Sweden lacks a national consensus for the organisation and provision of specialised palliative care.^46,47^ For this study, this implies an uncertainty regarding the definition of a palliative care consultation and what it entails, which varies between regions. Palliative care consultations may range from hospital consultation services with no patient responsibility to consultation services with some degree of medical responsibility supporting patients at home.

Another limitation stems from the Swedish Register of Palliative Care only representing care provided in the last week of life. This means we can only draw conclusions about consultations for this period. However, it is conceivable that similar patterns exist prior to the final week. Moreover, the coverage of the Swedish Register of Palliative Care varies between different types of care services, as well as between regions. However, there is no known systematic discrimination of specific patients.

## Conclusion

This general population study shows associations between palliative care consultations in the last week of life and factors related to disease, patient demographics and healthcare organisation. Patients with multiple symptoms, dying of neoplasms and those dying at home seem more likely to receive a palliative care consultation, while older people or those dying from other diseases might not access palliative care consultations to the same extent. This implies a need for policymakers, healthcare providers and societal measures to ensure equal access to palliative care. Further research investigating the underlying relationships between these factors, as well as other possible explanatory factors, is warranted.

## Supplemental Material

sj-docx-1-pcr-10.1177_26323524241293818 – Supplemental material for Palliative care consultation in the last week of life and associated factors: a cross-sectional general population study Supplemental material, sj-docx-1-pcr-10.1177_26323524241293818 for Palliative care consultation in the last week of life and associated factors: a cross-sectional general population study by Susanna Böling, Hanna Gyllensten, My Engström, Emma Lundberg, Johan Berlin and Joakim Öhlén in Palliative Care and Social Practice

sj-xlsx-2-pcr-10.1177_26323524241293818 – Supplemental material for Palliative care consultation in the last week of life and associated factors: a cross-sectional general population study Supplemental material, sj-xlsx-2-pcr-10.1177_26323524241293818 for Palliative care consultation in the last week of life and associated factors: a cross-sectional general population study by Susanna Böling, Hanna Gyllensten, My Engström, Emma Lundberg, Johan Berlin and Joakim Öhlén in Palliative Care and Social Practice

## Declarations

**Ethics approval and consent to participate:** The study was ethically assessed by the Swedish Ethical Review Authority (Dnr. 2019-05213). Since all participants in the study were deceased, the Ethical Review Authority stated that the study could be conducted without ethical vetting.

**Consent for publication:** Not applicable.

**Availability of data and materials:** The data for this study is available from each register holder. Certain restrictions may apply. Programming codes are available from the authors upon reasonable request.
