# Supplementary material for: Palliative care consultation in the last week of life and associated factors: a cross-sectional general population study
Source: Palliat Care Soc Pract. 2024 Nov 8;18:26323524241293818. doi: 10.1177/26323524241293818 (PMC11549695; doi:10.1177/26323524241293818)
Supplement: sj-docx-1-pcr-10.1177_26323524241293818 – Supplemental material for Palliative care consultation in the last week of life and associated factors: a cross-sectional general population study [file sj-docx-1-pcr-10.1177_26323524241293818.docx]

## Online-only Supplements

Palliative consultation in the last week of life and associated factors: a cross-sectional general-population study

# Authors

Susanna Böling^1^, Hanna Gyllensten^1^, My Engström^1,2^, Emma Lundberg^1^, Johan Berlin^3^, Joakim Öhlén^1,4,5^

# Author affiliations

1. Institute of Health and Care Sciences, Sahlgrenska Academy, University of Gothenburg, Gothenburg, Sweden. 2. Department of Surgery, Region Västra Götaland, Sahlgrenska University Hospital, Gothenburg, Sweden. 3. Department of Social and Behavioural Studies, University West, Trollhättan, Sweden. 4. Centre for Person-centred Care, University of Gothenburg, Gothenburg, Sweden. 5. Palliative Centre, Sahlgrenska University Hospital, Region Västra Götaland, Sweden.

**Contents**

[Online-only Supplements 1](#_Toc157630445)

[Authors 1](#_Toc157630446)

[Author affiliations 1](#_Toc157630447)

[eTable 1 – Source register information 3](#_Toc157630448)

[eFigure 1. Flowchart inclusion study population 6](#_Toc157630449)

[eTable 2. Multivariable logistic regression model with sociodemographic variables – factors associated with consultation of a palliative consultation service in last week of life on imputed data 7](#_Toc157630450)

[eTable 3. Multivariable logistic regression model with disease- and care-related variables – factors associated with consultation of a palliative consultation service in last week of life on imputed data 9](#_Toc157630451)

[eTable 4. Multivariable logistic regression model with care structure-related variables – factors associated with consultation of a palliative consultation service in last week of life on imputed data 10](#_Toc157630452)

[eTable 5. Multivariable logistic regression model with all variables – factors associated with consultation of a palliative consultation service in last week of life on imputed data 11](#_Toc157630453)

[eTable 6. Univariable logistic regression model with all variables – factors associated with consultation of a palliative consultation service in last week of life on all available data (no imputation) 13](#_Toc157630454)

[eTable 7. Multivariable logistic regression model – core set of explanatory variables (Multivariable Model 1 all available data) – factors associated with consultation of a palliative consultation service in last week of life on all available data (no imputation) 16](#_Toc157630455)

[eTable 8. Classification of Region of birth 18](#_Toc157630456)

[eTable 9. Underlying cause of death grouping (ICD-10) 19](#_Toc157630457)

# eTable 1 – Source register information

| The Swedish Register of Palliative Care | This national quality register is validated (Martinsson, Heedman, Lundström, & Axelsson, 2017) and contains information regarding care received in the last week of patients’ lives, as reported by the health care personnel responsible for their care in the unit where they died (Lundström et al., 2012). Patients, regardless of where they are cared for, can be reported in the register (including specialised and general palliative care services) (Swedish Register of Palliative Care, 2022). For the years 2013 through 2019 the register covered between 60 – 68 % of all deaths in Sweden (Swedish Register of Palliative Care, 2013, 2014, 2015, 2016, 2018, 2019, 2022). |
| --- | --- |
| The Swedish national cause of death register | The Swedish national cause of death register contains information regarding for example place of death, death date and underlying cause of death, as reported by a physician on the death certificate. The register is held by the National Board of Health and Welfare. Reliability and coverage are very good (Socialstyrelsen, 2022a). Coverage for 2013-2019 varied between 93-96% (Statistics Sweden, 2022a). Quality of measurement of for example underlying cause of death is dependent of the individual physician (Socialstyrelsen, 2022a), and discrepancies between what is reported on the death certificate and in the medical record may exist (Johansson, Björkenstam, & Westerling, 2009). Coding of causes of death is done by the National Board of Health and Welfare (Socialstyrelsen, 2022a). |
| The national patient register | The National patient register is held by the National Board of Health and Welfare. It is required that all specialised health care providers (public and private) report all hospitalisations and outpatient visits (to physicians) in the register. Reliability and measurement are considered good and coverage high, although, it is not possible with a complete control of coverage rate. Some quality problems exist regarding acute and planned visits to health care (Socialstyrelsen, 2022b) |
| The total population register (TPR) | The total population register is held by Statistics Sweden. The information which forms the basis for the register is retrieved from the Swedish Tax Agency (Skatteverket) and the Statistics Sweden's Real Property Register. The reliability of the register is considered good. However, for example deficiencies in self-reported change of residence may affect variables related to civil registration address. Uncertainty due to data management is considered small due to routines and control mechanisms (Statistics Sweden, 2022b). |
| The education register | Information regarding highest level of education achieved for Swedish residents is held by the Education register. Data is gathered from internal and external sources, public authorities and individual questionnaires to foreign born immigrants. The data is coded according to the SUN-classification, and reliability of the coding as well as the register itself is considered good, although a higher degree of uncertainty is seen among foreign born residents, especially recently immigrated (Statistics Sweden, 2022c). |
| The historic population register | The Historic population register contains information regarding demographic events like births, immigration and civil status for all registered residents in Sweden. It receives information from The total population register (TRP) and is held by Statistics Sweden (Statistics Sweden, 2005). |
| The multi-generation register | The multi-generation register contains information regarding country of birth and parentage (parents’ country of birth) for Swedish residents born after 1931 and registered as residents from 1961, with almost no individuals missing. Data is mainly retrieved from The Total population register. Measurement errors are minimal, and for data management manual procedures are few. Uncertainties due to re-use of personal identification numbers exists for a minority of residents (23 000) (Statistics Sweden, 2022d). |
|  |  |
| **References:** | Johansson, L. A., Björkenstam, C., & Westerling, R. (2009). Unexplained differences between hospital and mortality data indicated mistakes in death certification: an investigation of 1,094 deaths in Sweden during 1995. *Journal of Clinical Epidemiology, 62*(11), 1202-1209. |
|  | Lundström, S., Axelsson, B., Heedman, P. A., Fransson, G. & Fürst, C. J. (2012). Developing a national quality register in end-of-life care: The Swedish experience. Palliative Medicine, 26(4), 313-321. doi:10.1177/0269216311414758 |
|  | Martinsson, L., Heedman, P. A., Lundström, S. & Axelsson, B. (2017). Improved data validity in the Swedish Register of Palliative Care. PloS One, 12(10), e0186804. |
|  | Socialstyrelsen. (2022a). Det statistiska registrets framställning och kvalitet: Dödsorsaksregistret. Report no. 2022-5-7909. Retrieved from https://www.socialstyrelsen.se/globalassets/sharepoint-dokument/artikelkatalog/statistik/2022-5-7909.pdf [Accessed 3 August 2023]. |
|  | Socialstyrelsen. (2022b). Det statistiska registrets framställning och kvalitet: Patientregistret. Report no. 2022-2-7767. Retrieved from https://www.socialstyrelsen.se/globalassets/sharepoint-dokument/artikelkatalog/statistik/2022-2-7767.pdf [Accessed 3 August 2023]. |
|  | Statistics Sweden. (2005) Historiska befolkningsregistret (HBR) [Historic Population Register]. ISSN 1650-4917 Retrieved from https://libris.kb.se/bib/9958206 [Accessed 3 August 2023]. |
|  | Statistics Sweden. (2022a) [Webpage] https://www.scb.se [Accessed 15 November 2022] |
|  | Statistics Sweden. (2022b). Det Statistiska Registrets Framställning och Kvalitet - Registret över totalbefolkningen (RTB). Report no. BE0102. Retrieved from https://www.scb.se/contentassets/cab460c364b747cd9a4402aebdafc740/be0102_star_2021.pdf [Accessed 3 August 2023]. |
|  | Statistics Sweden. (2022c). Det Statistiska Registrets Framställning och Kvalitet - Registret över befolkningens utbildning. Report no. UF0537. Retrieved from https://www.scb.se/contentassets/450ea2ef5395452d8b150e69cf94e4f6/uf0537_ds_2021_v1_20220513.pdf [Accessed 3 August 2023]. |
|  | Statistics Sweden. (2022d). Det Statistiska Registrets Framställning och Kvalitet - Flergenerationsregistret 2021. Report no. BE0102. Retrieved from https://www.scb.se/contentassets/8dfda0b71365499c81dc27297bcb5f78/be0102_star_2021.pdf [Accessed 3 August 2023]. |
|  | Swedish Register of Palliative Care. (2013). Årsrapport för Svenska Palliativregistret verksamhetsåret 2013. Retrieved from https://palliativregistret.se/media/1mvpfgfz/%C3%A5rsrapport-2013.pdf [Accessed 3 August 2023]. |
|  | Swedish Register of Palliative Care. (2014). Årsrapport för Svenska Palliativregistret verksamhetsåret 2014. Retrieved from https://palliativregistret.se/media/hc0m5yl0/%C3%A5rsrapport-2014.pdf [Accessed 3 August 2023]. |
|  | Swedish Register of Palliative Care. (2015). Årsrapport för Svenska Palliativregistret verksamhetsåret 2015. Retrieved from https://palliativregistret.se/media/yp3jvj3g/a-rsrapport-2015.pdf [Accessed 3 August 2023]. |
|  | Swedish Register of Palliative Care. (2016). Årsrapport för Svenska Palliativregistret verksamhetsåret 2016. Retrieved from https://palliativregistret.se/media/rbtkayoq/%C3%A5rsrapport-2016.pdf [Accessed 3 August 2023]. |
|  | Swedish Register of Palliative Care. (2018). Årsrapport för Svenska palliativregistret 2018. Retrieved from https://palliativregistret.se/media/jqcaqz3i/%C3%A5rsrapport-2018.pdf [Accessed 3 August 2023]. |
|  | Swedish Register of Palliative Care. (2019). Årsrapport för Svenska palliativregistret 2019. Retrieved from https://palliativregistret.se/media/sjqh2ctm/a-rsrapport-2019.pdf [Accessed 3 August 2023]. |
|  | Swedish Register of Palliative Care. (2022). Svenska palliativregistret. [Webpage]. Retrieved from https://palliativregistret.se/ [Accessed 3 August 2023]. |

# eFigure 1. Flowchart inclusion study population

**Missing for each incl/excl and outcome variables in the population registered in the Swedish Register of Palliative Care**

Age n = 0
Expected death n = 1 352
Enrolled in a specialised palliative care service at time of death n = 0
Palliative consultation reported as ’don’t know’ n = 16 104

**Palliative consultation yes/no**

Yes n = 21 812
No n = 243 317

**Number of the study population reported in the Swedish Register of Palliative Care as Place of death ’other’ (for this study ’enrolled in a specialised palliative care service at time of death’ (yes/no))
n = 1 706**

**Number of the study population reported as Expected death ’don’t know’ n = 9 449**

**Source population**

**n = 602 209**

(Data from Statistics Sweden, National Board of Health and Welfare, and Swedish palliative care register)

Not registered in the Swedish Palliative Care Register n = 210 807 (35 %)

**Population registered in the Swedish Register of Palliative Care**

**n = 391 402**

Excluded with reasons
- Age <18 n = 505
- Unexpected death incl. missing n = 44 931
- Enrolled in a specialised palliative care service at time of death (yes) n = 65 349

- Palliative consultation reported as don’t know = 15 488

**Study population**

**n = 265 129**

# eTable 2. Multivariable logistic regression model with sociodemographic variables – factors associated with consultation of a palliative consultation service in last week of life on imputed data

| **Variable** | **Value** | **OR^a^ (95%CI)^b^** | **Pr > Chi-Square** | **Variable p-value** |
| --- | --- | --- | --- | --- |
| **Sex** | Male | 1 |  | 0.15 |
|  | Female | 1.02 (0.99-1.05) | 0.15 |  |
| **Age at death** | 80-89 | 1 |  | <.0001 |
|  | 18-29 | 4.02 (2.91-5.56) | <.0001 |  |
|  | 30-39 | 5.24 (4.19-6.56) | <.0001 |  |
|  | 40-49 | 5.62 (4.96-6.38) | <.0001 |  |
|  | 50-59 | 4.68 (4.35-5.04) | <.0001 |  |
|  | 60-69 | 3.27 (3.11-3.43) | <.0001 |  |
|  | 70-79 | 2.09 (2.02-2.17) | <.0001 |  |
|  | 90+ | 0.45 (0.43-0.47) | <.0001 |  |
| **Region of birth** | Sweden | 1 |  | 0.0006 |
|  | Nordic countries other than Sweden | 0.91 (0.85-0.97) | 0.0036 |  |
|  | EU28 other than the Nordic countries | 1.03 (0.94-1.12) | 0.56 |  |
|  | Outside EU28 and Nordic countries | 0.88 (0.80-0.96) | 0.0031 |  |
| **Marital status** | Married | 1 |  | <.0001 |
|  | Unmarried | 0.74 (0.70-0.78) | <.0001 |  |
|  | Widow | 0.87 (0.83-0.92) | <.0001 |  |
|  | Divorced | 0.88 (0.84-0.92) | <.0001 |  |
| **Educational attainment** | Higher secondary education | 1 |  | 0.0003 |
|  | No formal or elementary education | 0.96 (0.93-0.99) | 0.012 |  |
|  | Lower secondary education | 0.90 (0.85-0.95) | 0.0002 |  |
|  | Higher education | 1.00 (0.96-1.05) | 0.94 |  |
| **Have children under the age of 18** | No children under the age of 18 |  |  | 0.011 |
|  | Have children under the age of 18 | 0.89 (0.82-0.97) | 0.011 |  |
| **Living situation** | Owned residence | 1 |  | <.0001 |
|  | Rented residence | 0.93 (0.89-0.96) | <.0001 |  |
|  | Nursing home | 0.29 (0.28-0.31) | <.0001 |  |
|  | Other | 0.69 (0.62-0.77) | <.0001 |  |
| **Living in a single-person household** | Multi-person household |  |  | 0.0043 |
|  | Single-person household | 1.06 (1.02-1.11) | 0.0043 |  |
| **Residing in an urban area** | Not residing in an urban area |  |  | <.0001 |
|  | Residing in an urban area | 0.80 (0.77-0.83) | <.0001 |  |
| Area under ROC-curve with 95% CI for multivariable model = 0.74 (0.74-0.75) | | | | |
| ^a.^ Odds Ratio. ^b.^ Confidence Interval | | | | |

# eTable 3. Multivariable logistic regression model with disease- and care-related variables – factors associated with consultation of a palliative consultation service in last week of life on imputed data

| **Variable** | **Value** | **OR^a^ (95%CI)^b^** | **Pr > Chi-Square** | **Variable p-value** |
| --- | --- | --- | --- | --- |
| **Place of death** | Hospital | 1 |  | <.0001 |
|  | Nursing home | 1.59 (1.53-1.66) | <.0001 |  |
|  | Home | 4.09 (3.87-4.31) | <.0001 |  |
|  | Other place or unknown | 2.26 (1.76-2.92) | <.0001 |  |
| **Underlying cause of death** | Diseases of the circulatory system | 1 |  | <.0001 |
|  | Neoplasms | 9.54 (9.10-10.00) | <.0001 |  |
|  | Diseases of the digestive system | 1.80 (1.60-2.02) | <.0001 |  |
|  | Diseases of the nervous system | 2.68 (2.40-2.98) | <.0001 |  |
|  | Diseases of the respiratory system | 1.29 (1.18-1.41) | <.0001 |  |
|  | Endocrine, nutritional and metabolic diseases | 1.38 (1.21-1.57) | <.0001 |  |
|  | Infectious diseases | 1.24 (1.08-1.44) | 0.0027 |  |
|  | Dementia^c^ | 0.71 (0.64-0.78) | <.0001 |  |
|  | Other | 1.22 (1.10-1.34) | <.0001 |  |
| **Number of days in the reporting care service (categorical)** | 0-2 | 1 |  | <.0001 |
|  | 3-7 | 1.22 (1.15-1.30) | <.0001 |  |
|  | 8-30 | 1.56 (1.47-1.66) | <.0001 |  |
|  | 31-182 | 1.28 (1.20-1.37) | <.0001 |  |
|  | 183-365 | 0.76 (0.70-0.83) | <.0001 |  |
|  | 366- | 0.47 (0.43-0.50) | <.0001 |  |
| **Number of hospital transfers in last month of life** |  | 1.08 (1.06-1.10) | <.0001 | <.0001 |
| **Symptom presence in last week of life** | No reported symptoms | 1 |  | <.0001 |
|  | yes, one symptom | 1.39 (1.28-1.51) | <.0001 |  |
|  | yes, two symptoms | 1.81 (1.68-1.96) | <.0001 |  |
|  | yes, three or more symptoms | 2.54 (2.35-2.73) | <.0001 |  |
| Area under ROC-curve with 95% CI for multivariable model = 0.85 (0.84-0.85) | | | | |
| ^a.^ Odds Ratio ^b.^ Confidence Interval ^c.^ Including senility | | | | |

# eTable 4. Multivariable logistic regression model with care structure-related variables – factors associated with consultation of a palliative consultation service in last week of life on imputed data

| **Variable** | **Value** | **OR^a^ (95%CI)^b^** | **Pr > Chi-Square** | **Variable p-value** |
| --- | --- | --- | --- | --- |
| **Year of death** |  | 1.02 (1.01-1.03) | <.0001 | <.0001 |
| **Health care region** | South region | 1 |  | <.0001 |
|  | Southeast region | 1.32 (1.26-1.38) | <.0001 |  |
|  | West region | 1.32 (1.26-1.38) | <.0001 |  |
|  | Stockholm region | 0.32 (0.29-0.34) | <.0001 |  |
|  | Uppsala-Örebro region | 0.92 (0.88-0.96) | <.0001 |  |
|  | North region | 0.98 (0.93-1.03) | 0.42 |  |
| Area under ROC-curve with 95% CI for multivariable model = 0.58 (0.58-0.59) | | | | |
| ^a.^ Odds Ratio. ^b.^ Confidence Interval | | | | |

# eTable 5. Multivariable logistic regression model with all variables – factors associated with consultation of a palliative consultation service in last week of life on imputed data

| **Variable** | **Value** | **OR^a^ (95%CI)^b^** | **Pr > Chi-Square** | **Variable p-value** |
| --- | --- | --- | --- | --- |
| **Year of death** |  | 1.03 (1.02-1.04) | <.0001 | <.0001 |
| **Place of death** | Hospital |  |  | <.0001 |
|  | Nursing home | 1.84 (1.76-1.92) | <.0001 |  |
|  | Home | 3.68 (3.49-3.89) | <.0001 |  |
|  | Other place or unknown | 2.53 (1.95-3.27) | <.0001 |  |
| **Sex** | Male | 1 |  | 0.0049 |
|  | Female | 1.05 (1.01-1.08) | 0.0049 |  |
| **Age at death** | 80-89 | 1 |  | <.0001 |
|  | 18-29 | 2.35 (1.63-3.38) | <.0001 |  |
|  | 30-39 | 2.77 (2.16-3.56) | <.0001 |  |
|  | 40-49 | 2.80 (2.43-3.22) | <.0001 |  |
|  | 50-59 | 2.40 (2.21-2.60) | <.0001 |  |
|  | 60-69 | 1.86 (1.76-1.96) | <.0001 |  |
|  | 70-79 | 1.46 (1.40-1.52) | <.0001 |  |
|  | 90+ | 0.63 (0.59-0.66) | <.0001 |  |
| **Region of birth** | Sweden | 1 |  | 0.43 |
|  | Nordic countries other than Sweden | 1.02 (0.95-1.09) | 0.63 |  |
|  | EU28 other than the Nordic countries | 1.08 (0.98-1.19) | 0.11 |  |
|  | Outside EU28 and Nordic countries | 0.99 (0.90-1.09) | 0.80 |  |
| **Underlying cause of death** | Diseases of the circulatory system | 1 |  | <.0001 |
|  | Neoplasms | 7.03 (6.70-7.39) | <.0001 |  |
|  | Diseases of the digestive system | 1.46 (1.30-1.64) | <.0001 |  |
|  | Diseases of the nervous system | 1.84 (1.65-2.05) | <.0001 |  |
|  | Diseases of the respiratory system | 1.20 (1.10-1.31) | <.0001 |  |
|  | Endocrine, nutritional and metabolic diseases | 1.17 (1.03-1.34) | 0.020 |  |
|  | Infectious diseases | 1.18 (1.02-1.36) | 0.027 |  |
|  | Dementia^c^ | 0.68 (0.61-0.74) | <.0001 |  |
|  | Other | 1.18 (1.07-1.30) | 0.0007 |  |
| **Marital status** | Married | 1 |  | 0.0052 |
|  | Unmarried | 0.90 (0.85-0.96) | 0.0006 |  |
|  | Widow | 0.98 (0.93-1.03) | 0.41 |  |
|  | Divorced | 0.97 (0.92-1.03) | 0.34 |  |
| **Number of days in the reporting care service (categorical)** | 0-2 | 1 |  | <.0001 |
|  | 03-07 | 1.24 (1.16-1.32) | <.0001 |  |
|  | 08-30 | 1.56 (1.47-1.66) | <.0001 |  |
|  | 31-182 | 1.30 (1.21-1.40) | <.0001 |  |
|  | 183-365 | 0.87 (0.79-0.95) | 0.0022 |  |
|  | 366- | 0.60 (0.55-0.65) | <.0001 |  |
| **Educational attainment** | Higher secondary education | 1 |  | 0.026 |
|  | No formal or elementary education | 0.96 (0.92-0.99) | 0.022 |  |
|  | Lower secondary education | 0.93 (0.88-0.99) | 0.016 |  |
|  | Higher education | 0.99 (0.94-1.04) | 0.73 |  |
| **Health care region** | South region | 1 |  | <.0001 |
|  | Southeast region | 1.27 (1.20-1.34) | <.0001 |  |
|  | West region | 1.24 (1.18-1.30) | <.0001 |  |
|  | Stockholm region | 0.47 (0.43-0.51) | <.0001 |  |
|  | Uppsala-Örebro region | 0.86 (0.82-0.91) | <.0001 |  |
|  | North region | 1.12 (1.06-1.19) | 0.0001 |  |
| **Have children under the age of 18** | No children under the age of 18 | 1 |  | 0.34 |
|  | Have children under the age of 18 | 0.95 (0.86-1.05) | 0.34 |  |
| **Living situation** | Owned residence | 1 |  | <.0001 |
|  | Rented residence | 0.97 (0.93-1.01) | 0.14 |  |
|  | Nursing home | 0.75 (0.70-0.80) | <.0001 |  |
|  | Other | 0.88 (0.79-0.99) | 0.030 |  |
| **Number of hospital transfers in last month of life** |  | 1.05 (1.02-1.07) | <.0001 | <.0001 |
| **Living in a single-person household** | Multi-person household | 1 |  | 0.19 |
|  | Single-person household | 0.97 (0.93-1.02) | 0.19 |  |
| **Symptom presence in last week of life** |  | 1.33 (1.30-1.35) | <.0001 | <.0001 |
| **Residing in an urban area** | Not residing in an urban area | 1 |  | <.0001 |
|  | Residing in an urban area | 0.88 (0.84-0.92) | <.0001 |  |
| Area under ROC-curve with 95% CI for multivariable model = 0.85 (0.85-0.86) | | | | |
| ^a.^ Odds Ratio ^b.^ Confidence Interval ^c.^ Including senility | | | | |

# eTable 6. Univariable logistic regression model with all variables – factors associated with consultation of a palliative consultation service in last week of life on all available data (no imputation)

| **Variable** | **n missing** | **Value** | **n (%) of event** | **OR^a^ (95%CI)^b^** | **p-value** | **Area under ROC-Curve (95%CI)** |
| --- | --- | --- | --- | --- | --- | --- |
|  |  |  |  | **Consultation with a palliative service** |  |  |
| **Sex** | 0 | **Male** | 11135 (9.6%) |  |  |  |
|  |  | **Female** | 10677 (7.1%) | 0.72 (0.70-0.74) | <0.0001 | 0.54 (0.54-0.54) |
| **Age at death continous (OR per 10 units)** | 0 | **18-113** | 21812 (8.2%) | 0.53 (0.52-0.53) | <0.0001 | 0.73 (0.72-0.73) |
| **Age at death** | 0 | **80-89** | 7148 (6.7%) | 1.00 | <0.0001*** |  |
|  |  | **18-29** | 49 (21.9%) | 3.90 (2.84-5.36) | <0.0001 |  |
|  |  | **30-39** | 114 (27.7%) | 5.35 (4.31-6.65) | <0.0001 |  |
|  |  | **40-49** | 426 (30.5%) | 6.12 (5.44-6.87) | <0.0001 |  |
|  |  | **50-59** | 1304 (27.4%) | 5.27 (4.92-5.64) | <0.0001 |  |
|  |  | **60-69** | 3626 (21.3%) | 3.77 (3.61-3.94) | <0.0001 |  |
|  |  | **70-79** | 6816 (14.5%) | 2.36 (2.28-2.44) | <0.0001 |  |
|  |  | **90+** | 2329 (2.7%) | 0.38 (0.36-0.40) | <0.0001 | 0.71 (0.70-0.71) |
| **Region of birth** | 5 | **Sweden** | 19462 (8.1%) | 1.00 | <0.0001*** |  |
|  |  | **Nordic countries other than Sweden** | 1104 (8.2%) | 1.00 (0.94-1.07) | 0.92 |  |
|  |  | **EU28 other than the Nordic countries** | 580 (8.9%) | 1.10 (1.01-1.20) | 0.026 |  |
|  |  | **Outside EU28 and Nordic countries** | 666 (10.8%) | 1.37 (1.26-1.48) | <0.0001 | 0.51 (0.50-0.51) |
| **Educational attainment** | 5449 | **Higher secondary education** | 8725 (9.9%) | 1.00 | <0.0001*** |  |
|  |  | **No formal or elementary education** | 7829 (6.4%) | 0.63 (0.61-0.65) | <0.0001 |  |
|  |  | **Lower secondary education** | 1870 (9.7%) | 0.98 (0.93-1.03) | 0.43 |  |
|  |  | **Higher education** | 3067 (10.1%) | 1.02 (0.98-1.06) | 0.39 | 0.56 (0.55-0.56) |
| **Marital status** | 24 | **Married** | 9530 (12.1%) | 1.00 | <0.0001*** |  |
|  |  | **Unmarried** | 2636 (9.9%) | 0.81 (0.77-0.84) | <0.0001 |  |
|  |  | **Widow** | 5966 (4.9%) | 0.38 (0.36-0.39) | <0.0001 |  |
|  |  | **Divorced** | 3676 (9.7%) | 0.79 (0.76-0.82) | <0.0001 | 0.61 (0.61-0.62) |
| **Living in a single-person household** | 591 | **Multi-person household** | 12447 (11.2%) |  |  |  |
|  |  | **Single-person household** | 9316 (6.1%) | 0.51 (0.49-0.52) | <0.0001 | 0.58 (0.58-0.59) |
| **Have children under the age of 18** | 591 | **No children under the age of 18** | 20978 (8.1%) |  |  |  |
|  |  | **Have children under the age of 18** | 785 (14.6%) | 1.95 (1.80-2.10) | <0.0001 | 0.51 (0.51-0.51) |
| **Living situation** | 15505 | **Owned residence** | 13519 (11.7%) | 1.00 | <0.0001*** |  |
|  |  | **Rented residence** | 5709 (10.0%) | 0.85 (0.82-0.88) | <0.0001 |  |
|  |  | **Nursing home** | 1614 (2.3%) | 0.18 (0.17-0.19) | <0.0001 |  |
|  |  | **Other** | 384 (7.0%) | 0.57 (0.51-0.63) | <0.0001 | 0.63 (0.63-0.63) |
| **Residing in an urban area** | 22 | **Not residing in an urban area** | 3734 (13.2%) |  |  |  |
|  |  | **Residing in an urban area** | 18074 (7.6%) | 0.54 (0.52-0.56) | <0.0001 | 0.54 (0.53-0.54) |
| **Underlying cause of death** | 0 | **Diseases of the circulatory system** | 2196 (2.5%) | 1.00 | <0.0001*** |  |
|  |  | **Neoplasms** | 16591 (26.1%) | 13.99 (13.37-14.65) | <0.0001 |  |
|  |  | **Diseases of the digestive system** | 359 (4.8%) | 2.00 (1.78-2.24) | <0.0001 |  |
|  |  | **Diseases of the nervous system** | 423 (5.8%) | 2.44 (2.20-2.72) | <0.0001 |  |
|  |  | **Diseases of the respiratory system** | 652 (3.3%) | 1.37 (1.26-1.50) | <0.0001 |  |
|  |  | **Endocrine, nutritional and metabolic diseases** | 258 (3.4%) | 1.38 (1.21-1.58) | <0.0001 |  |
|  |  | **Infectious diseases** | 216 (3.0%) | 1.21 (1.05-1.40) | 0.0077 |  |
|  |  | **Dementia^c^** | 580 (1.3%) | 0.51 (0.47-0.56) | <0.0001 |  |
|  |  | **Other** | 537 (3.1%) | 1.26 (1.14-1.38) | <0.0001 | 0.80 (0.80-0.81) |
| **Place of Death** | 0 | **Hospital** | 8048 (8.4%) | 1.00 | <0.0001*** |  |
|  |  | **Nursing home** | 8834 (5.9%) | 0.69 (0.67-0.71) | <0.0001 |  |
|  |  | **Home** | 4838 (24.5%) | 3.54 (3.40-3.69) | <0.0001 |  |
|  |  | **Other place or unknown** | 92 (20.6%) | 2.84 (2.25-3.57) | <0.0001 | 0.61 (0.61-0.62) |
| **Number of hospital transfers in last month of life** | 0 | **0-<1** | 7923 (6.1%) |  |  |  |
|  |  | **1-10** | 13889 (10.3%) | 1.27 (1.25-1.28) | <0.0001 | 0.57 (0.57-0.58) |
| **Number of hospital transfers in last month of life (categorical)** | 0 | **None** | 7923 (6.1%) | 1.00 | <0.0001*** |  |
|  |  | **One transfer** | 8791 (10.1%) | 1.74 (1.69-1.80) | <0.0001 |  |
|  |  | **Two transfers** | 3744 (11.0%) | 1.91 (1.84-1.99) | <0.0001 |  |
|  |  | **Three or more transfers** | 1354 (10.5%) | 1.82 (1.71-1.93) | <0.0001 | 0.57 (0.57-0.58) |
| **Number of days in the reporting care service** | 161 | **0-2** | 1672 (6.1%) |  |  |  |
|  |  | **3-7** | 3344 (9.3%) |  |  |  |
|  |  | **8-30** | 7846 (14.5%) |  |  |  |
|  |  | **31-182** | 5348 (15.0%) |  |  |  |
|  |  | **183-365** | 1131 (6.8%) |  |  |  |
|  |  | **366-** | 2459 (2.6%) | 0.81 (0.80-0.82) | <0.0001 | 0.61 (0.60-0.61) |
| **Symptom presence in last week of life** | 0 | **No reported symptoms** | 852 (3.1%) |  |  |  |
|  |  | **yes, one symptom** | 2696 (5.1%) |  |  |  |
|  |  | **yes, two symptoms** | 5734 (7.5%) |  |  |  |
|  |  | **yes, three or more symptoms** | 12530 (11.6%) | 1.59 (1.56-1.61) | <0.0001 | 0.61 (0.61-0.62) |
| **Health care region** | 22 | **South region** | 3942 (8.3%) | 1.00 | <0.0001*** |  |
|  |  | **Southeast region** | 3900 (10.7%) | 1.32 (1.26-1.38) | <0.0001 |  |
|  |  | **West region** | 5643 (10.7%) | 1.32 (1.26-1.38) | <0.0001 |  |
|  |  | **Stockholm region** | 930 (2.8%) | 0.32 (0.30-0.34) | <0.0001 |  |
|  |  | **Uppsala-Örebro region** | 5024 (7.7%) | 0.92 (0.88-0.96) | <0.0001 |  |
|  |  | **North region** | 2369 (8.1%) | 0.98 (0.93-1.03) | 0.42 | 0.58 (0.58-0.58) |
| **Year of Death (continous)** | 0 | **2013** | 2957 (7.7%) |  |  |  |
|  |  | **2014** | 3152 (8.1%) |  |  |  |
|  |  | **2015** | 3146 (8.1%) |  |  |  |
|  |  | **2016** | 3175 (8.2%) |  |  |  |
|  |  | **2017** | 3257 (8.5%) |  |  |  |
|  |  | **2018** | 3083 (8.5%) |  |  |  |
|  |  | **2019** | 3042 (8.6%) | 1.02 (1.01-1.03) | <0.0001 | 0.51 (0.51-0.51) |
| All tests are performed with univariable logistic regression. | | | | | | |
| P-values, OR and Area under ROC-curve are based on original values and not on stratified groups. | | | | | | |
| OR is the ratio for the odds for an increase of the predictor of one unit. | | | | | | |
| ***) p-value for the entire effect/factor/variable. | | | | | | |
| ^a.^ Odds Ratio ^b.^ Confidence Interval ^c.^ Including senility | | | | | | |

# eTable 7. Multivariable logistic regression model – core set of explanatory variables (Multivariable Model 1 all available data) – factors associated with consultation of a palliative consultation service in last week of life on all available data (no imputation)

| **Variable** | **Value** | **OR^a^ (95%CI)^b^** | **Pr > Chi-Square** | **Variable p-value** |
| --- | --- | --- | --- | --- |
| **Year of death** |  | 1.03 (1.02-1.04) | <.0001 | <.0001 |
| **Place of death** | Hospital | 1 |  | <.0001 |
|  | Nursing home | 1.39 (1.34-1.45) | <.0001 |  |
|  | Home | 2.87 (2.74-3.01) | <.0001 |  |
|  | Other place or unknown | 2.42 (1.87-3.14) | <.0001 |  |
| **Sex** | Male | 1 |  | 0.086 |
|  | Female | 1.03 (1.00-1.06) | 0.086 |  |
| **Living in a single-person household** | Multi-person household | 1 |  | <.0001 |
|  | Single-person household | 0.86 (0.83-0.89) | <.0001 |  |
| **Age at death** | 80-89 | 1 |  | <.0001 |
|  | 18-29 | 2.15 (1.45-3.17) | 0.0001 |  |
|  | 30-39 | 2.46 (1.90-3.17) | <.0001 |  |
|  | 40-49 | 2.67 (2.33-3.05) | <.0001 |  |
|  | 50-59 | 2.37 (2.19-2.56) | <.0001 |  |
|  | 60-69 | 1.90 (1.80-2.00) | <.0001 |  |
|  | 70-79 | 1.52 (1.46-1.58) | <.0001 |  |
|  | 90+ | 0.56 (0.53-0.59) | <.0001 |  |
| **Region of birth** | Sweden | 1 |  | 0.55 |
|  | Nordic countries other than Sweden | 0.98 (0.92-1.06) | 0.68 |  |
|  | EU28 other than the Nordic countries | 1.07 (0.97-1.19) | 0.17 |  |
|  | Outside EU28 and Nordic countries | 1.00 (0.90-1.11) | 0.98 |  |
| **Underlying cause of death** | Diseases of the circulatory system | 1 |  | <.0001 |
|  | Neoplasms | 8.56 (8.15-8.99) | <.0001 |  |
|  | Diseases of the digestive system | 1.64 (1.46-1.84) | <.0001 |  |
|  | Diseases of the nervous system | 1.63 (1.46-1.82) | <.0001 |  |
|  | Diseases of the respiratory system | 1.24 (1.13-1.35) | <.0001 |  |
|  | Endocrine, nutritional and metabolic diseases | 1.10 (0.96-1.26) | 0.16 |  |
|  | Infectious diseases | 1.23 (1.07-1.42) | 0.0043 |  |
|  | Dementia^c^ | 0.52 (0.47-0.57) | <.0001 |  |
|  | Other | 1.22 (1.10-1.34) | <.0001 |  |
| **Educational attainment** | Higher secondary education | 1 |  | 0.0015 |
|  | No formal or elementary education | 0.94 (0.91-0.98) | 0.0021 |  |
|  | Lower secondary education | 0.92 (0.87-0.98) | 0.0055 |  |
|  | Higher education | 1.00 (0.95-1.05) | 0.95 |  |
| **Health care region** | South region | 1 |  | <.0001 |
|  | Southeast region | 1.29 (1.22-1.36) | <.0001 |  |
|  | West region | 1.26 (1.20-1.32) | <.0001 |  |
|  | Stockholm region | 0.46 (0.42-0.50) | <.0001 |  |
|  | Uppsala-Örebro region | 0.89 (0.84-0.93) | <.0001 |  |
|  | North region | 1.13 (1.06-1.20) | <.0001 |  |
| **Symptom presence in last week of life** |  | 1.35 (1.32-1.37) | <.0001 | <.0001 |
| Area under ROC-curve with 95% CI for multivariable model = 0.85 (0.84-0.85) | | | | |
| ^a.^ Odds Ratio ^b.^ Confidence Interval ^c.^ Including senility | | | | |

# eTable 8. Classification of Region of birth

| **Sweden** |
| --- |
| **Nordic countries other than Sweden** |
| Norway, Denmark, Denmark, Finland, Iceland |
| **EU28 other than the Nordic countries** |
| Belgium, Bulgaria, France, Estonia, Cyprus, Italy, Ireland, Greece, Croatia, Latvia, Malta, Lithuania, Luxembourg, Poland, Portugal, Romania, Netherlands, Slovakia, Slovenia, Spain, Czech Republic, United Kingdom of Great Britain and Northern Ireland, Germany, Hungary, Austria, Poland, Czechoslovakia, Germany |
| **Outside EU28 and Nordic countries** |
| **Europe other than EU28 and Nordic countries:** Serbia and Montenegro, Belarus, Holy See, Yugoslavia, Ukraine, Turkey, Switzerland, Serbia, San Marino, Russian Federation, Montenegro, Republic of Moldova, Monaco, North Macedonia, Liechtenstein, Kosovo, Gibraltar, Bosnia and Herzegovina, Albania, Andorra. **Africa:** Algeria, Angola, Benin, Botswana, Burkina Faso, Burundi, Central African Republic, Comoros, Djibouti, Egypt, Equatorial Guinea, Cote d'Ivoire, Eritrea, Ethiopia, Gabon, Gambia, Ghana, Guinea, Guinea-Bissau, Cameroon, Cape Verde, Kenya, Congo, Congo, the Democratic Republic of the, Lesotho, Liberia, Libya, Madagascar, Malawi, Mali, Mauritania, Mauritius, Morocco, Mozambique, Namibia, Niger, Nigeria, Rwanda, Sao Tome and Principe, Senegal, Seychelles, Sierra Leone, Somalia, Sudan, Eswatini, South Africa, South Sudan, United Republic of Tanzania, Chad, Togo, Tunisia, Uganda, Egypt, Zambia, Zimbabwe, Morocco. **North America:** Anguilla, Antigua and Barbuda, Belize, Barbados, Bahamas, British Virgin Islands, Bermuda, Costa Rica, Grenada, Guatemala, Jamaica, Haiti, Honduras, El Salvador, Dominica, Dominican Republic, Canada, Cuba, Mexico, Nicaragua, Panama, Saint Kitts and Nevis, Saint Lucia, Saint Vincent and the Grenadines, Trinidad and Tobago, United States. **South America:** Suriname, Venezuela, Uruguay, Paraguay, Peru, Argentina, Chile, Colombia, Bolivia, Brazil, Ecuador, Guyana. **The former Soviet Union. Asia & Oceania:** Vietnam, State of Palestine, Timor-Leste, Malaysia, Hong Kong, India, Yemen, Vietnam, Uzbekistan, Turkmenistan, Thailand, Syrian Arab Republic, Tajikistan, Taiwan, Sri Lanka, Singapore, Saudi Arabia, Qatar, Oman, Pakistan, Nepal, Mongolia, Lebanon, Myanmar, Malaysia, Maldives, Kuwait, Lao People's Democratic Republic, Democratic People's Republic of Korea, Republic of Korea, China, Kyrgyzstan, Kazakhstan, Japan. Yemen, Jordan, Cambodia, Georgia, India, Indonesia, Iraq, Iran, Israel, Philippines, United Arab Emirates, Brunei Darussalam, Bhutan, Afghanistan, Armenia, Azerbaijan, Bahrain, Bangladesh, Fiji, Australia, New Zealand, Papua New Guinea, Palau, Nauru, Federated States of Micronesia, Marshall Islands, Kiribati, Vanuatu, Tonga, Tuvalu, Samoa, Solomon Islands, Samoa. |

# eTable 9. Underlying cause of death grouping (ICD-10)

| **Underlying cause of death** | **ICD-10 starting with** |
| --- | --- |
| Neoplasm | C, D0, D1, D2, D3, D4 |
| Diseases of the circulatory system | I |
| Diseases of the digestive system | K |
| Diseases of the nervous system | G (except G30) |
| Diseases of the respiratory system | J |
| Endocrine, nutritional and metabolic diseases | E |
| Infectious diseases | A, B |
| Dementia, including senility | G30, R54, F01, F03, F05 |
| Other (including mental and behavioural disorders, and external causes of morbidity and mortality) | F (except F01, F03, F05), V, W, X, Y |
